# Influences on patient satisfaction in healthcare centers: a semi-quantitative study over 5 years

**DOI:** 10.1186/s12913-017-2307-z

**Published:** 2017-05-19

**Authors:** Ruth D. Thornton, Nicole Nurse, Laura Snavely, Stacey Hackett-Zahler, Kenice Frank, Robert A. DiTomasso

**Affiliations:** 1 Department of Psychology, Philadelphia College of Osteopathic Medicine, 4170 City Ave., Philadelphia, PA 19131 USA; 2 North Philadelphia Health System/St. Joseph’s Hospital, Philadelphia, PA USA; 3 Geisinger Medical Center, Danville, PA USA; 4 Maria Fareri Children’s Hospital, Westchester, NY USA; 5 College of Podiatric Medicine & Surgery, Des Moines University, Des Moines, IO USA

**Keywords:** Patient satisfaction, Health care delivery, Community health

## Abstract

**Background:**

Knowledge of ambulatory patients’ satisfaction with clinic visits help improve communication and delivery of healthcare. The goal was to examine patient satisfaction in a primary care setting, identify how selected patient and physician setting and characteristics affected satisfaction, and determine if feedback provided to medical directors over time impacted patient satisfaction.

**Methods:**

A three-phase, semi-quantitative analysis was performed using anonymous, validated patient satisfaction surveys collected from 889 ambulatory outpatients in 6 healthcare centers over 5-years. Patients’ responses to 21 questions were analyzed by principal components varimax rotated factor analysis. Three classifiable components emerged: Satisfaction with Physician, Availability/Convenience, and Orderly/Time. To study the effects of several independent variables (location of clinics, patients’ and physicians’ age, education level and duration at the clinic), data were subjected to multivariate analysis of variance (MANOVA)..

**Results:**

Changes in the healthcare centers over time were not significantly related to patient satisfaction. However, location of the center did affect satisfaction. Urban patients were more satisfied with their physicians than rural, and inner city patients were less satisfied than urban or rural on Availability/Convenience and less satisfied than urban patients on Orderly/Time.

How long a patient attended a center most affected satisfaction, with patients attending >10 years more satisfied in all three components than those attending <1–5 years. Level of education affected patients’ satisfaction only in the component Orderly/Time; patients without a high school education were significantly less satisfied than those with more. Patients in their 40′s were significantly less satisfied in Availability/Convenience than those >60 years old.

Patients were significantly more satisfied with their 30–40 year-old physicians compared with those over 60. On Orderly/Time, patients were more satisfied with physicians who were in their 50′s than physicians >60.

**Conclusions:**

Improvement in patient satisfaction includes a need for immediate, specific feedback. Although Medical Directors received feedback yearly, we found no significant changes in patient satisfaction over time. Our results suggest that, to increase satisfaction, patients with lower education, those who are sicker, and those who are new to the center likely would benefit from additional high quality interactions with their physicians.

**Electronic supplementary material:**

The online version of this article (doi:10.1186/s12913-017-2307-z) contains supplementary material, which is available to authorized users.

## Background

Patient satisfaction surveys are often used to understand patients’ concerns and determine areas for improvement, including improving communication between physicians and patients. Survey results document progress and allow physicians and staff to maintain high standards. Although results of patient satisfaction surveys are used by payer systems to profile individual physicians and guide physician compensation, one study showed that < 25% of primary care physicians found profiles useful for improving patient care and fewer used the profiles to change [1]. Improvements are more likely to occur if staff receives more immediate feedback [2].

Data collection methods play a role in outcomes. On-site surveys provide an immediate outlet for patients who are experiencing problems, although higher ratings for on-site surveys may also relate directly to doctor-patient communication. Surveys administered later after a clinic visit may yield lower ratings, possibly due to the course of treatment [2, 3].

Many factors influence patient satisfaction. Patient demographics such as age, gender, income, socioeconomic and general health status impact patients’ responses [3, 4]. Characteristics of the medical provider, including demographics and experience, also affect their interactions with patients [5–9]. Other factors include the type of setting the patient is in [10] and the amount of time patients had to wait [11]. However, Anderson found that complaints about wait time can be moderated by time spent with the physician [12].

Physician characteristics extend beyond the obvious. Physician-patient concordance in race, gender or age may be important in patient satisfaction, but many other factors such as primary language, parental status, sexual orientation, values, beliefs, or communication style may be associated [13, 14]. How long the patient has been with this physician and the degree to which the physicians’ communication is patient-centered are significant [13]. A physician’s experience plays a role, with lowest patient satisfaction with first-year residents; interestingly, residents with some more experience attained similar satisfaction ratings to those of the faculty attendings, suggesting that the requisite skills are acquired during the first year of training [7].

Whether to administer patient satisfaction surveys depends on the overall goals of the medical facility and on physician buy-in to change [1, 15]. The views of the medical director and administrator are key components as to whether the surveys are taken seriously and acted upon by physicians [16]. Patient satisfaction can become a success criterion of the center when physicians and staff meet regularly to discuss improvements in a context of cooperation and mutual support.

## Methods

We initiated this study of patient satisfaction to help physicians better understand their patients at the healthcare centers (HCCs) of a not-for-profit medical school’s outpatient primary care centers on the east coast. Physicians were provided raw data and results of open-ended questions very soon after each year’s study. However, we decided to statistically analyze the overall data in order to understand where patients were most and least satisfied and what influenced their satisfaction. Our goal was to provide information which could help focus physician directors’ changes to improve patient satisfaction.

The research was under the auspices of a medical college (Philadelphia College of Osteopathic Medicine, PCOM) which owns and operates five outpatient HCCs, four of which are located within the city limits of Philadelphia and the fifth HCC located in a rural area. [17] Two within Philadelphia are considered urban, while two are in the inner city [18]. An additional nonaffiliated, inner city HCC located within Philadelphia was also used in the research. We considered the nonaffiliated HCC as a control, but expected it to likely agree with data from the affiliated inner city HCCs. The quantity of surveys administered are listed in Table 1.

**Table 1:** Numbers of patients surveyed from each Healthcare center during year 1 (Fall, 2005), year 2 (Summer, 2007), and year 3 (Summer 2010)

HCC	Location	Year 1 # surveyed	Year 2 # surveyed	Year 3 # surveyed	TOTAL surveyed
1	Inner City (PCOM)	40	68	90	198
2	Urban (PCOM)	34	54	69	157
3	Inner City (PCOM)	21	43	70	134
4	Rural (PCOM)	30	19	25	74
5	Urban (PCOM)	25	51	45	121
6	Inner City (non-PCOM)	50	75	80	205
TOTALS		200	310	379	889

This research arose from a need to quickly and inexpensively conduct patient satisfaction surveys in the Healthcare Centers, incorporating a research component involving graduate students interested in health related careers. Surveys were administered to patients at the five HCCs. Patient questions were adapted from the validated *DiTomasso-Willard Patient Satisfaction Questionnaire* [19] (questions are listed in Table 2). Demographic information and responses to open-ended questions were also collected. In 2005 (year 1), 2007 (year 2), and 2010 (year 3), students in a master’s program at the medical school approached patients in the waiting areas at each HCC asking them to complete a survey. Patients could take the surveys with them into the examination room, but they returned the survey before leaving the HCC. If requested, the student helped a patient read the questions.

**Table 2:** Grouping of the 21 survey questions using factor analysis, Rotated Component Matrix

	Component:		
Question:	1	2	3
Q1. During a typical visit, my doctor spends enough time explaining my medical condition to me.	*0.773*	0.151	0.162
Q2. My doctor gives me the best quality of care.	*0.869*	0.190	0.133
Q3. I would recommend my doctor to friends.	*0.827*	0.191	0.112
Q4. The staff are helpful to the patients.	0.311	*0.562*	0.127
Q5. My doctor uses technical terms that confuse me.^a^	0.109	−0.200	*0.628*
Q6. My doctor is available when I need him/her.^b^	0.412	0.491	0.076
Q7. The waiting room time is too long.^b^	−0.084	0.402	0.442
Q8. My doctor really follows through.	*0.751*	0.230	0.082
Q9. I plan to return to this center for care.	*0.713*	0.370	0.148
Q10. It’s easy to get an appointment when I need one.	0.223	*0.665*	0.122
Q11. My doctor wastes time talking about things that don’t really matter to me.^a^	0.271	−0.014	*0.702*
Q12. My doctor treats the “whole” person.	*0.640*	0.300	0.143
Q13. The staff accommodates my needs over the phone.	0.241	*0.677*	0.070
Q14. I am satisfied with the quality of the medical care I receive here.	*0.724*	0.398	0.171
Q15. I receive prompt attention while waiting in this facility.	0.285	*0.658*	0.132
Q16. I have to tell my story several times before getting an appointment.^a^	0.001	0.409	*0.631*
Q17. I am treated the same as other people who get care here.	0.366	*0.511*	0.105
Q18. Check-out time at the front desk is too time-consuming.^a^	−0.030	0.338	*0.648*
Q19. I would not recommend this center to a friend.^a^	0.250	0.101	*0.530*
Q20. Everything seems so confusing at this center.^a^	0.199	0.160	*0.731*
Q21. When I’m sick I can get an appointment pretty quickly.	0.229	*0.712*	0.057

Component 1: Satisfaction with Doctor (Questions 1, 2, 3, 8, 9, 12, 14)

Component 2: Availability/Convenience (Questions 4, 10, 13, 15, 17, 20)

Component 3: Orderly/Time (Questions 5, 11, 16, 18, 19, 20)

^a^Questions worded in the negative were reversed for statistical analysis

^b^Question not classified by component

Each surveying period was conducted over an approximately one month of time. Students varied their sampling by time of day and day of week. Therefore, the sample was comprised of a random representation of patients attending each HCC during each one-month period of surveying. The students approached anyone who was in the waiting room during sampling times, but patients were free to refuse if they wished. The goal was to obtain approximately 10% of the average number of patients seen by each HCC in a month.

The protocol (Protocol #H05-022X) was approved by the Institutional Review Board (IRB) of PCOM that determined it to be exempt from informed consent requirements under 45 CFR 46.101(b)(2)--survey research in which the responses will be recorded in such a manner that the human subjects cannot be identified, directly or through identifiers linked to the subjects (e.g., name, Social Security number). Further, no master list existed linking such identifiers to the subjects. Approximately 5–15% of the average numbers of patients coming to each HCC in a month were surveyed. Inclusion criteria included patients willing to respond, patient age of at least 18 years, and patients who spoke English. Patients were assured the questionnaire was confidential without any identifying information, the results would be presented in aggregate form, and that their responses would not affect their specific care at the HCC. In order to maintain anonymity, a patient’s medical status was not requested, although in retrospect, it may have been helpful. From observation, students reported that those with acute medical issues were less inclined to participate. Although an absolute count was not performed, students who administered surveys consistently estimated that only about 5% of the patients in the waiting room refused to participate.

Survey results were entered into IBM’s Statistical Package for the Social Sciences (SPSS 18.0) for analysis. Missing data were filled in using Linear Interpolation, and any negative questions were transformed to the positive on the Likert scale, so that, for all questions, 5 (strongly agree) meant “most satisfied.” All 21 survey statements were subjected to a principal components varimax rotated factor analysis according to Kaiser’s criterion [20] which ultimately allowed for a reduction of statements into three classifiable components, Satisfaction with Physician, Availability/Convenience, and Orderly/Time (Table 2).

Following each survey period, the data were analyzed in SPSS to collapse the questions into three classifiable components/categories. These three categories did not vary during the 3 data collection periods. After each survey period, study staff attended face-to-face meetings with Medical Directors of each healthcare center, the Dean of the Medical School, and the Chair of Family Medicine to present the results. HCC staff were provided with mean scores for each question for their HCC compared with a composite of all HCC’s. They also received the data collapsed into the three categories for their HCC compared with a composite of all HCC’s, but without statistical analysis.

For analysis of the composite data, multivariate analysis of variance (MANOVA) was performed for groups of data, using post hoc Tukey to distinguish specific significance between groups. Independent *t*-test was used for gender analysis, and Chi square analysis was done to compare the observed gender data from patients who completed surveys with patient demographics of each HCC. See Additional Data for more specific information.

In using factor analysis, it is common practice to require 10 subjects per number of items. In the present case, this criterion was far exceeded. For the separate MANOVA analyses using 3 dependent variables, setting power at 95% for a medium effect size at the 0.05 level of significance comparing 2 levels (male vs. female) of the independent variable, 3 levels (3 locations) and 5 levels (physician age groups), the required number of subjects was 280, 171, and 145 respectively. In all cases there was sufficient power.

## Results

Surveys were administered to a total of 889 patients who visited one of the HCCs for treatment (Table 1). These numbers represented between 5–15% of the average number of patients seen monthly in the affiliated HCCs, and comparable numbers of surveys were obtained from the much larger, non-affiliated HCC.

Applying principal components varimax rotated factor analysis to the survey responses resulted in groups of identifiable questions that constituted factors (Rotated component matrix for all questions is shown on Table 2). Three classifiable factors, Satisfaction with Physician, Availability/Convenience, and Orderly/Time, emerged from the analysis and are used throughout this research. Two questions (Q6 and Q7) were not included as the items did not load on any of the factors (Table 2). Using the survey questions that constituted each factor (Table 2), the three factors have the following characteristics: Satisfaction with Physician involves being satisfied with the quality of medical care received, as well as the physician spending enough time with the patient. Availability/Convenience involves being satisfied with the staff and their helpfulness in making appointments, whether in person or by phone. Orderly/Time has to do with patients’ time being respected, and interactions with staff and physicians being clear and to the point, avoiding confusion.

Overall, patients were quite satisfied with their HCCs, as evidenced by overall mean scores greater than 3.89 on a Likert scale of 1–5 (see Additional file 1: Table S3A). Mean scores were highest in Satisfaction with Physician (4.27 ± 0.65), while Availability/Convenience (3.92 ± 0.69) and Orderly/Time (3.89 ± 0.66) were somewhat lower. Even so, a score of 3.9 represents the top 20–25% of satisfaction. The open-ended responses emphasized the importance of patients’ satisfaction with their physician, even if patients were somewhat less satisfied with other aspects of their visit (see Additional file 2: Table S6).

The goal of this research was to identify areas found to be statistically significant. More complete data can be found in the Additional files 1, 2, 3,and 4. Based on MANOVA, there was no significance over time in any of the three categories (see Additional file 1: Table S3B). This points to a consistency over time in the operations and functioning of these HCC’s.

**Fig. 1:**
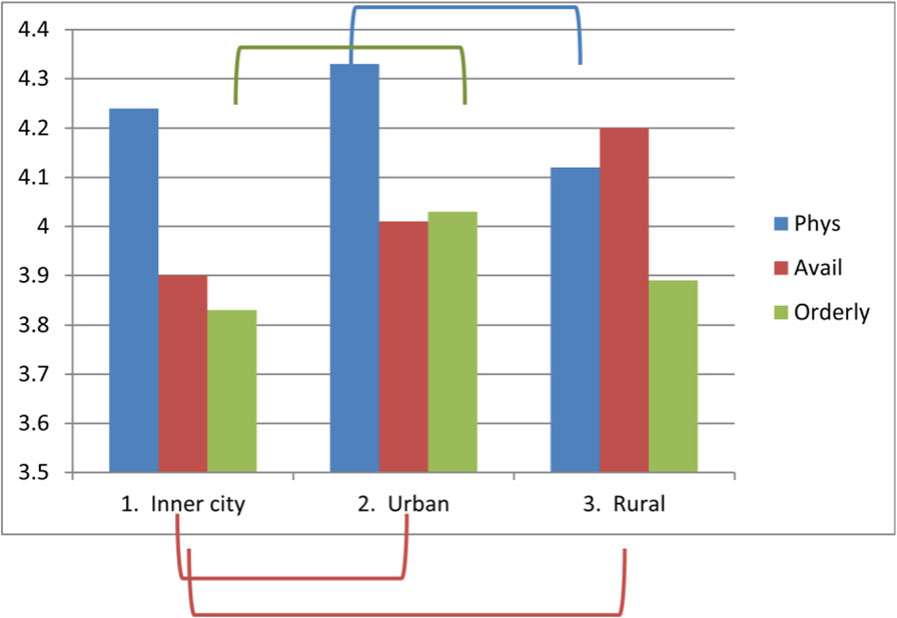
Satisfaction by location (Inner City, Urban and Rural). Lines/Brackets indicate comparisons by color that were significantly different in each of the categories
- When individual HCCs were analyzed (Fig. 2), one urban HCC (#5) had significantly higher satisfaction with their Physician than the other urban HCC (#2) or one inner city HCC (#6). The other urban HCC (#2) had more satisfaction in the category of Orderly/Time than two of the three inner city HCCs (#3 and #6). Two inner city HCCs (#1 and #6) had significantly lower satisfaction in the category of Availability/Convenience than the rural HCC (#4). (See Additional file 1: Table S3D, for details.)

The following areas were found to be statistically significant by MANOVA:

- Analyzing satisfaction in inner city, urban and rural HCCs (Fig. 1), significance was observed in the following area.: Patients in inner city HCCs were less satisfied than those in urban or rural HCC’s on Availability/Convenience, and those in inner city HCCs were less satisfied than urban patients in the area of Orderly/Time. Urban patients were more satisfied with their Physician than were rural patients while inner city patients’ satisfaction with their Physician was not significantly different from the other localities (See Additional file 1: Table S3C, for more detail).

**Fig. 2:**
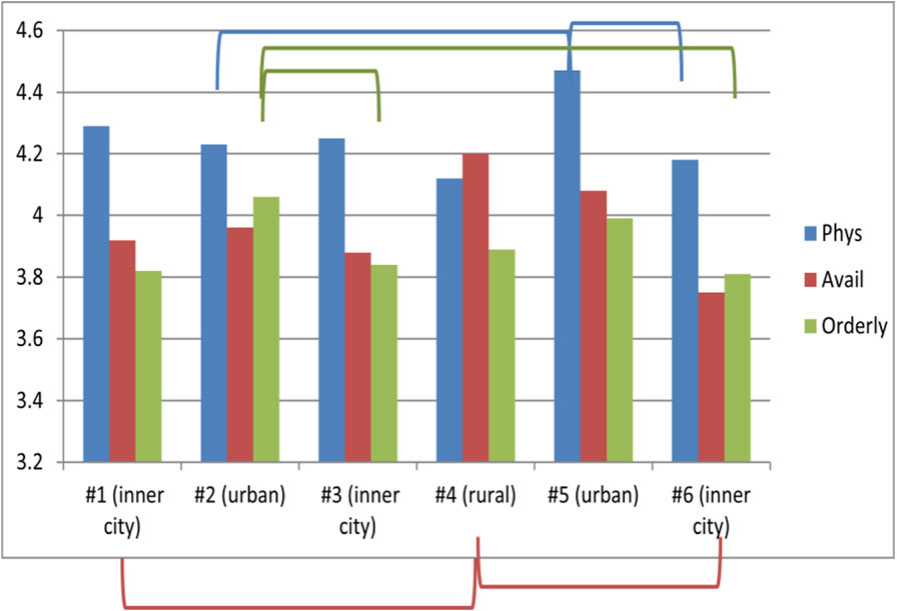
Satisfaction by individual HCCs. Lines/Brackets indicate comparisons by color that were significantly different in each of the categories
- Patients’ demographics appear to play a role in the level of satisfaction. Patients over 60 years old were more satisfied with the Availability/Convenience of the HCC than patients who were in their 40′s (Fig. 3). Those with more education (in the range from graduating high school through graduate school) were more satisfied with the Orderly/Time category than those with less than a high school diploma (Fig. 4). Finally, patients who had been with their HCC for longer periods of time were more satisfied than those who had been there less than 5 years in all three categories of satisfaction with Physician, Availability/Convenience, and Orderly/Time (Fig. 5) (See Additional file 2: Table S4C, for details).

**Fig. 3:**
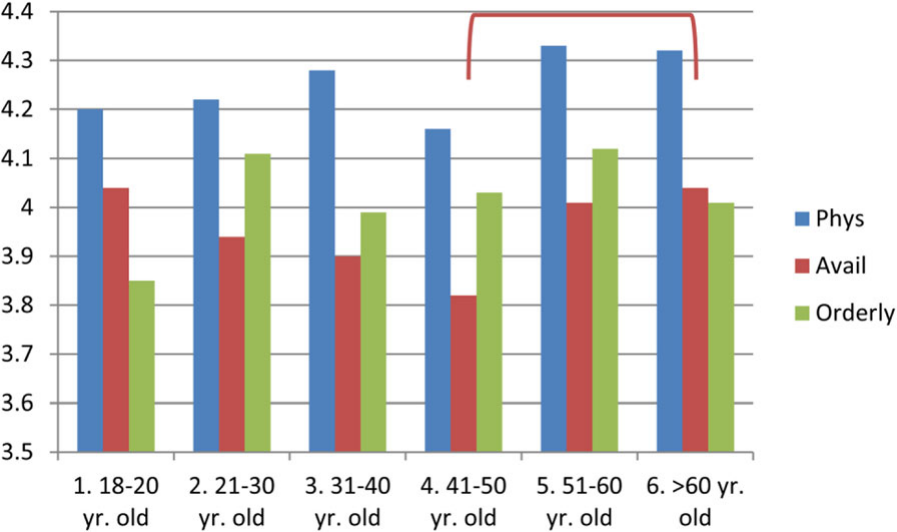
The effect of patient age on satisfaction. Lines/Brackets indicate comparisons by color that were significantly different in each of the categories

**Fig. 4:**
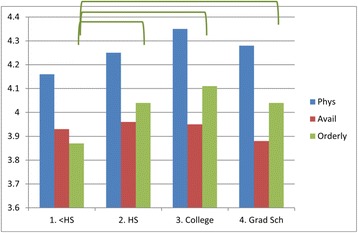
The effect of patient education on satisfaction. Lines/Brackets indicate comparisons by color that were significantly different in each of the categories

**Fig. 5:**
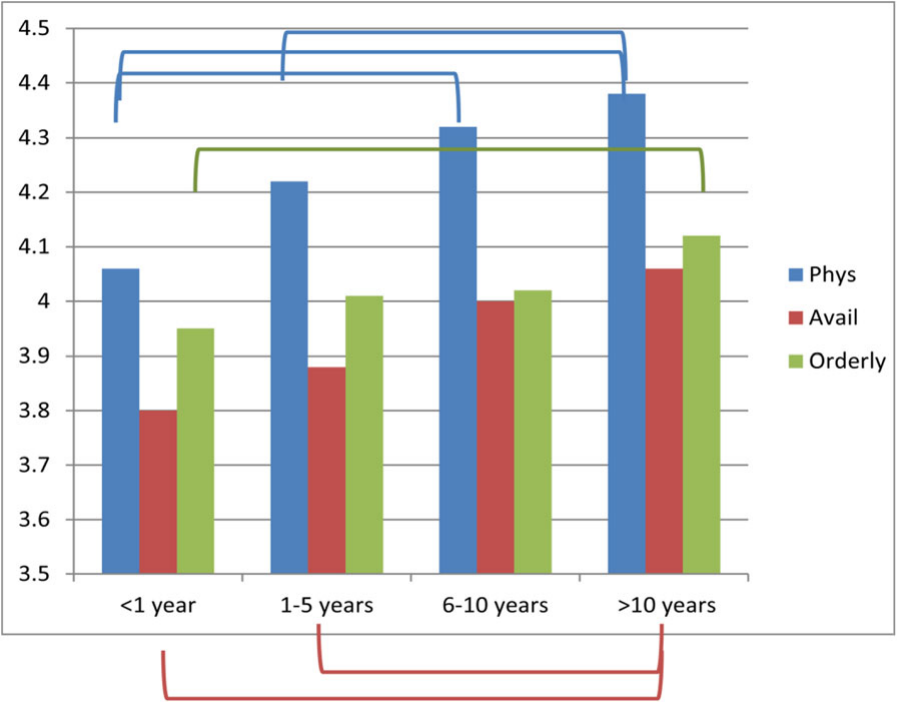
The effect of length of time at a HCC on satisfaction. Lines/Brackets indicate comparisons by color that were significantly different in each of the categories
- Physicians in these centers tended to longevity in their positions. Patients were more satisfied with their Physicians who were in their 30′s and 40′s than with physicians in their 50′s (Fig. 6). Also, physicians in their 50′s were perceived to be more Available than those in their 60′s. Patients rated male physicians as more Available than female physicians, and in the Inner City HCCs, patients rated their Caucasian physicians higher on Availability than African American physicians (see Additional file 3: Table S5B and C).

**Fig. 6:**
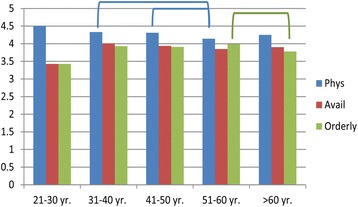
The effect of physician’s age on satisfaction. Lines/Brackets indicate comparisons by color that were significantly different in each of the categories
- Open ended responses were overall very positive, with the exception of the rural HCC4 during year 1. After personnel replacements at this HCC, more positive responses were also seen there. Wait times were seen as a problem in some HCCs, particularly in the inner city centers. The majority of patients were very satisfied with the convenience of their HCC (See Additional file 4: Table S6).

## Discussion

In the examination of changes over time, patient satisfaction at the HCCs in the study remained overall quite high in all three categories of Satisfaction with Physician, Availability/Convenience, and Orderly/Time. Meanwhile, notable changes at the affiliated centers during this time period included a new telephone system installed between years 1–2, major renovations of one of the centers in year 2, and installation of a system of Electronic Medical Records (EMR) in all affiliated HCCs between years 2 and 3; the non-affiliated HCC #6 also introduced EMR prior to year 3. While telephone changes would likely affect staff-patient interactions, instituting EMR represented a major change in the physician-patient interactions, with the addition of computers to each examination room. We were surprised that these seemingly “major” changes did not significantly affect the satisfaction levels over this time period. De Leon et al. found generally higher patient satisfaction with a center after EMR were introduced [21], while we found no significant differences after EMR was installed.

Results of the patient satisfaction surveys were presented to Medical Directors and staff in a timely manner after each survey period, but without statistical analysis. From the initial data given to each HCC, medical staff could compare their mean results with a composite mean result for all the centers. However, they did not have access to comparisons of individual HCCs (see Additional file 1, Table S3D). Nor did they have access to figures such as Fig. 2, comparing individual HCCs. It is not surprising that each HCC is unique. An example is HCC 5 with a significantly higher level of satisfaction with Physician compared with two other HCCs, one urban and inner city (Fig. 2, and Additional file 1: Table S3D). This merits more in-depth analysis of the physician practices at this outstanding urban HCC as a positive example for others.

We projected that HCCs sharing similar locations (inner city, urban or rural) would be more alike and this proved to be the case. In the components of Availability/Convenience and Orderly/Time, patients in the inner city HCCs were less satisfied than those in urban or rural settings, consistent with findings of the individual HCCs. There could be several reasons for differences between inner city and other HCCs. Fan et al. found that functional status (disease severity, physical limitation) was only weakly associated with general satisfaction, while education, coping skills and disease perception were more important to patient’s satisfaction [4]. Patients in the inner city may be sicker due to overall inadequate health knowledge or reluctance to visit a doctor, possibly due to lack of insurance. These findings suggest that physician-patient interactions with the goal of improved disease understanding might help as much as actual improvement in health. We did not ask for the health status of individual patients in our survey, so we can only guess the health status of patients at different locations.

Comparing locations (Fig. 1) with individual HCCs (Fig. 2) reveals the sources of these differences. For example, in Fig. 1, inner city patients were statistically less satisfied in the component of Orderly/Time than were patients in urban settings. Fig. 2 shows that the differences were primarily with one urban HCC #2 (but not with urban HCC #5), compared only with 2 inner city HCC #3 and #6 (but not with inner city HCC #1). So generalizations require examining the individual HCCs as well.

Education level of the patient can also be reflective of location. Approximately 80% of inner city respondents reported having high school education or less, similar to rural patients (76%), while only 58% of urban patients had a high school education or less (data not shown). Other issues facing patients, such as availability of public transportation, may be more of an obstacle in the inner city than in either urban or rural settings. While public transportation is also not widely available in rural settings, it is likely most patients have access to a vehicle. Inner city respondents also were less satisfied in the component of Orderly/Time than respondents in urban settings, and this is confirmed in the open-ended questions (see Additional file 4: Table S6) where a larger number of patients specifically mentioned the wait time as a problem in the inner city HCCs than in the urban or rural HCCs. Although we wondered if dissatisfaction with wait time could be directly attributable to student participation in the examination room, that seems not to be the case, as a very small percent of responders mentioned students in the open-ended questions and half of those were positive. Mol et al. found that patients generally felt neutral or positive about the presence of students, and in that study, between 83 and 98% of patients consented to student participation [22].

Our only finding of differences associated with education level in satisfaction were in the area of Orderly/Time; patients with less than a high school education were less satisfied in the component of Orderly/Time than any other group. This could be due to their inability to understand the medical parlance or the protocols involved in their care. However, one study also found that the converse-a physician’s satisfaction with a patient-was associated with their patients’ higher education level [23], suggesting that the responsibility may be reciprocal between the physician and the patient.

Another patient demographic of age can also contribute to patient satisfaction. Our finding that patients over 60 years old had a higher degree of satisfaction in Availability/Convenience is not surprising. This finding agrees with Jackson who reported that patients over 65 years old and with higher functional status were more satisfied [3]. Peck found that physicians were more likely to have patient-centered encounters with patients over age 65, which in turn meant that older patients were more satisfied [24]. Although there was no impact of patients’ gender on level of satisfaction, we did find that, in general, more female patients agreed to fill out the surveys than were actually represented as patients in the HCCs. Not surprisingly, the most significant differences were found in the length of time a patient had been attending their HCC. This is undoubtedly a self-selection, where either the physician or the location suits the patient who continues to visit that center. Pelletier calls this “sampling bias,” citing that “those who stay with a program…may be those who are most satisfied” [23]. Another explanation is through “visit continuity,” where respondents rated the quality of physician-patient interaction as being more important during the early stages of continuity or when the patient reported worse self-rated health [25]. This suggests that physicians who focus on those newer patients or sicker patients who would benefit the most from additional interactions may have the most positive results over time.

Demographics of the physician may also be important to patient satisfaction. The physicians at the affiliated HCCs were all osteopathic (DO) physicians, who self-reported that they used Osteopathic Manipulative Treatment at their clinics about 20% of the time. In the open-ended questions, some patients did express a preference for DO physicians. On age of physicians, it appears that more patients prefer a physician younger than 50 years old in the component of Satisfaction with Physician, but in Orderly/Time, they prefer a physician in their 50′s rather than in their 60′s. We speculate that physicians in their 50′s are likely to be at the pinnacle of their profession, although other considerations may also be important, such as humor or degree of connection that the patient perceives with that physician. In the variable of Orderly/Time, it is possible that physicians in their 50′s may be more efficient, having a well-run visit, while the slower, possibly more thorough pace of older physicians may not be as appreciated.

The statistical significances found in this data enhance the details which were presented to the medical directors after each surveying period and provide additional measures of patient satisfaction. Presenting the data to medical directors in figure form rather than as graphs is likely to enhance understanding. Finally, presenting the data of each individual HCC rather than as a composite may help medical directors to see the larger picture.

The present study has several limitations: In retrospect from patients’ written responses, an additional choice under the education demographic would have better captured any additional education received, such as technical certificates or Associate degrees. Also, the severity of the patient’s medical condition should have been noted, as this has been shown to influence patient satisfaction [4]. In addition, the questions that fell under the component Orderly/Time in the factor analysis fortuitously contained all questions which had been originally stated in the negative and then were reversed for analysis. Finally, presentation of the data to the medical directors in a timely fashion could be improved by presenting figures in addition to tables, and showing results of each individual HCC.

## Conclusions

This study was designed to provide feedback to Medical Directors on patient satisfaction in their HCCs. Our findings point to a consistency in the operations and functioning of these HCCs over time, even when renovations or installation of EMR were performed. Differences in locality (inner city, urban, rural) were found, as well as differences in satisfaction by patient demographics (age, education level, length of time with a HCC) and by physician demographics (age, gender). However, uniqueness of individual HCCs contributes to these differences. Physicians from each HCC regularly meet together, and they can use these meetings to help better understand and build on their strengths and individuality. Results of this study can be used to increase satisfaction if physicians help their patients benefit from their services and increase their satisfaction. Particularly, physicians can concentrate on providing additional high-quality interactions for patients with less education, those who are sicker, and those who are new to the HCC.

## Additional files

Additional file 1: Table S3. Comparisons overall and by time, location, individual HCCs vs. 3 components. (DOC 41 kb)

Additional file 2: Table S4. Patient demographics vs. three factors. *refers to higher mean score; ns, not significant. (DOC 44 kb)

Additional file 3: Table S5. Physician demographics vs. three factors. *refers to higher mean score; ns, not significant. (DOC 40 kb)

Additional file 4: Table S6. Open-ended questions by healthcare center and year. (+) refers to positive statements, what did you like most? (−) refers to negative statements, what did you like least? (DOC 48 kb)

## Abbreviations

DO: Doctor of Osteopathic Medicine
EMR: Electronic Medical Records
HCCs: Healthcare Centers
IRB: Institutional Review Board
MANOVA: Multivariate analysis of variants
PCOM: Philadelphia College of Osteopathic Medicine
SPSS: Statistical Package for the Social Sciences
