# Supplementary material for: Influences on patient satisfaction in healthcare centers: a semi-quantitative study over 5 years
Source: BMC Health Serv Res. 2017 May 19;17:361. doi: 10.1186/s12913-017-2307-z (PMC5438500; doi:10.1186/s12913-017-2307-z)
Supplement: Supplementary file 1 — Comparisons overall and by time, location, individual HCCs vs. 3 components. (DOC 41 kb) [file 12913_2017_2307_MOESM1_ESM.doc]

**Additional File1.**

**Table S3.** Comparisons overall and by time, location, individual HCCs vs. 3 components

| **A. All HCCs** | **Sat. w/Phys** | **Avail./Conv** | **Orderly/Time** |
| --- | --- | --- | --- |
| Overall | 4.27±0.65 | 3.92±0.69 | 3.89±0.66 |
| **B. ALL HCC, by TIME:** | **Sat. w/Phys** | **Avail./Conv** | **Orderly/Time** |
| 1. Year 1 | 4.14 ±0.74 | 3.88±0.69 | 4.04±0.51 |
| 2. Year 2 | 4.30 ±0.57 | 3.97±0.68 | 4.07±0.68 |
| 3. Year 3 | 4.28 ±0.70 | 3.94±0.70 | 4.01±0.76 |
| **Wilks' λ=0.987,**  **F(6,1764)=1.926,**  **p=0.070** |  |  |  |
| Post-Hoc Tukey | ns | ns | ns |
| **C. ALL HCC, by LOCATION:** | **Sat. w/Phys** | **Avail./Conv** | **Orderly/Time** |
| 1. Inner city | 4.24±0.66 | 3.90±0.72 | 3.83±0.66 |
| 2. Urban | 4.33±0.65 | 4.01±0.68 | 4.03±0.69 |
| 3. Rural | 4.12±0.77 | 4.20±0.47 | 3.89±0.58 |
| **Wilks' λ=0.941, F(6,1764)=9.107, p=0.000** |  |  |  |
| Post-Hoc Tukey | 2* vs. 3, p=0.050 | 2* vs. 1, p=**0.003**  3* vs. 1, p=**0.000** | 2* vs. 1, p=**0.011** |
| **D. Individual HCCs** | **Sat. w/Phys** | **Avail./Conv** | **Orderly/Time** |
| 1. #1 (inner city) | 4.29±0.64 | 3.92±0.73 | 3.82±0.68 |
| 2. #2 (urban) | 4.23±0.71 | 3.96±0.68 | 4.06±0.64 |
| 3. #3 (inner city) | 4.25±0.64 | 3.88±0.70 | 3.84±0.62 |
| 4. #4 (rural) | 4.12±0.78 | 4.20±0.47 | 3.89±0.58 |
| 5. #5 (urban) | 4.47±0.52 | 4.08±0.66 | 3.99±0.74 |
| 6. #6 (inner city) | 4.18±0.69 | 3.75±0.68 | 3.81±0.62 |
| **Wilks' λ=0.924,**  **F(15,2415)=4.695,**  **p=0.000** |  |  |  |
| Post hoc Tukey | 5* vs. 2, p=**0.028**  5* vs. 6, p=**0.002** | 4* vs. 1, p=**0.044**  4* vs. 3, p=**0.024**  4* vs. 6; p=**0.000**  2* vs. 6, p=**0.038**  5* vs. 6, p=**0.000** | 2* vs. 1, p=**0.006**  2* vs. 3, p=**0.047**  2* vs. 6, p=**0.003** |

*refers to higher mean score; ns, not significant
