# Supplementary material for: Influences on patient satisfaction in healthcare centers: a semi-quantitative study over 5 years
Source: BMC Health Serv Res. 2017 May 19;17:361. doi: 10.1186/s12913-017-2307-z (PMC5438500; doi:10.1186/s12913-017-2307-z)
Supplement: Supplementary file 2 — Patient demographics vs. three factors. *refers to higher mean score; ns, not significant. (DOC 44 kb) [file 12913_2017_2307_MOESM2_ESM.doc]

**Additional File 7.**

**Table S4.** Patient demographics vs. three factors

| **A. ALL HCCs by AGE:** | **Sat. w/Phys** | **Avail./Conv** | **Orderly/Time** |
| --- | --- | --- | --- |
| 1. 18-20 yr. old | 4.20±0.68 | 4.04±0.72 | 3.85±0.83 |
| 2. 21-30 yr. old | 4.22±0.69 | 3.94±0.74 | 4.11±0.69 |
| 3. 31-40 yr. old | 4.28±0.62 | 3.90±0.63 | 3.99±0.64 |
| 4. 41-50 yr. old | 4.16±0.79 | 3.82±0.76 | 4.03±0.68 |
| 5. 51-60 yr. old | 4.33±0.62 | 4.01±0.72 | 4.12±0.62 |
| 6. >60 yr. old | 4.32±0.59 | 4.04±0.64 | 4.01±0.66 |
| **Wilks' λ=0.966,**  **F(15,2391)=1.99**  **p=0.013** |  |  |  |
| Post-Hoc Tukey | ns | 6* vs. 4, p=**0.019** | ns |
| **B. ALL HCCs by level education** | **Sat. w/Phys** | **Avail./Conv** | **Orderly/Time** |
| 1. <High School | 4.16±0.67 | 3.93±0.71 | 3.87±0.71 |
| 2. High School | 4.25±0.67 | 3.96±0.68 | 4.04±0.67 |
| 3. College | 4.35±0.68 | 3.95±0.77 | 4.11±0.69 |
| 4. Grad School | 4.28±0.66 | 3.88±0.68 | 4.04±0.56 |
| **Wilks' λ=0.973,**  **F(9,2105)=2.603**  **p=0.005** |  |  |  |
| Post-Hoc Tukey | ns | ns | 2* vs. 1, p=**0.029**  3* vs. 1, p=**0.010**  4* vs. 1, p=**0.011** |
| **C. ALL HCCs by length of time at center** | **Sat. w/Phys** | **Avail./Conv** | **Orderly/Time** |
| 1. <1 year | 4.06±0.78 | 3.80±0.78 | 3.95±0.72 |
| 2. 1-5 years | 4.22±0.69 | 3.88±0.70 | 4.01±0.66 |
| 3. 6-10 years | 4.32±0.56 | 4.00±0.60 | 4.02±0.64 |
| 4. >10 years | 4.38±0.60 | 4.06±0.68 | 4.12±0.65 |
| **Wilks' λ=0.967,**  **F(9,2136)=3.252**  **p=0.001** |  |  |  |
| Post-Hoc Tukey | 3* vs. 1, p=**0.003**  4* vs. 1, p=**0.000**  4* vs. 2, p=**0.026** | 4* vs. 1, p=**0.001**  4* vs. 2, p=**0.014** | 4* vs. 1, p=**0.032** |

*refers to higher mean score; ns, not significant
