# Supplementary material for: Influences on patient satisfaction in healthcare centers: a semi-quantitative study over 5 years
Source: BMC Health Serv Res. 2017 May 19;17:361. doi: 10.1186/s12913-017-2307-z (PMC5438500; doi:10.1186/s12913-017-2307-z)
Supplement: Supplementary file 3 — Physician demographics vs. three factors. *refers to higher mean score; ns, not significant. (DOC 40 kb) [file 12913_2017_2307_MOESM3_ESM.doc]

**Additional File 9.**

**Table S5.** Physician demographics vs. three factors

| **A. ALL HCCs by Physician Age** | **Sat. w/Phys** | **Avail./Conv** | **Orderly/Time** |
| --- | --- | --- | --- |
| 1. 21-30 yr. old (n=2) | 4.50±0.71 | 3.43±0.20 | 3.43±0.61 |
| 2. 31-40 yr. old | 4.33±0.64 | 4.01±0.70 | 3.93±0.73 |
| 3. 41-50 yr. old | 4.31±0.61 | 3.94±0.66 | 3.91±0.62 |
| 4. 51-60 yr. old | 4.14±0.78 | 3.85±0.75 | 3.99±0.59 |
| 5. >60 yr. old | 4.25±0.62 | 3.90±0.71 | 3.78±0.66 |
| **Wilks' λ=0.965,**  **F(9,1900)=3.120**  **p=0.001** |  |  |  |
| Post-Hoc Tukey | 2* vs. 4, p=**0.014**  3* vs. 4, p=**0.006** | ns | 4* vs. 5, p=**0.044** |
| **B. ALL HCCs by Gender** | **Sat. w/Phys** | **Avail./Conv** | **Orderly/Time** |
| 1. Physician male | 4.26±0.65 | 3.99±0.68 | 4.03±0.65 |
| 2. Physician female | 4.270.69± | 3.89±0.74 | 4.06±0.66 |
| **Wilks' λ=0.989,**  **F(3,785)=3.009**  **p=0.030** |  |  |  |
| Post-Hoc Tukey |  | 1* vs. 2, p=**0.044** |  |
| **C. ALL HCCs in inner city , phys. ethnicity:** | **Sat. w/Phys** | **Avail./Conv** | **Orderly/Time** |
| 1. Physician, Afr.Am | 4.35±0.62 | 3.89±0.74 | 3.85±0.67 |
| 2. Physician, Caucasian | 4.26±0.63 | 4.00±0.65 | 3.93±0.66 |
| **Wilks' λ=0.960,**  **F(3,670)=9.305**  **p=0.034** |  |  |  |
| Post hoc Tukey |  | 1vs.2*, p=**0.034** |  |

*refers to higher mean score; ns, not significant
