# Supplementary material for: Influences on patient satisfaction in healthcare centers: a semi-quantitative study over 5 years
Source: BMC Health Serv Res. 2017 May 19;17:361. doi: 10.1186/s12913-017-2307-z (PMC5438500; doi:10.1186/s12913-017-2307-z)
Supplement: Supplementary file 4 — Open-ended questions by healthcare center and year. (+) refers to positive statements, what did you like most? (−) refers to negative statements, what did you like least? (DOC 48 kb) [file 12913_2017_2307_MOESM4_ESM.doc]

**Additional File 2.**

**Table S6.** Open-ended questions by healthcare center and year

| HCC | Year | Total #. surveys | # (%) responses | Doctor  Pos, Neg | Staff  Pos, Neg | Wait time Pos, Neg | Apptm Pos, Neg | Convenience  Pos, Neg |
| --- | --- | --- | --- | --- | --- | --- | --- | --- |
| #1 | 1 | 40 | 38 (95%) | 48+, 4- | 22+, 2- | 4+, 26- | 1+, 2- | 16+, 1- |
| Inner | 2 | 68 | 63 (93%) | 37+, 4- | 17+, 9- | 7+, 14- | 1+, 4- | 14+, 2- |
| City | 3 | 90 | 78 (86%) | 48+, 4- | 22+, 2- | 4+, 26- | 1+, 2- | 16+, 1- |
| #2 | 1 | 34 | 31 (91%) | 21+, 4- | 14+, 5- | 2+, 8- | 1+, 3- | 6+, 2- |
| Urban | 2 | 54 | 47 (87%) | 32+, 4- | 21+, 2- | 8+, 7- | 2+, 7- | 6+, 1- |
|  | 3 | 69 | 61 (88%) | 33+, 9- | 14+, 3- | 5+, 5- | 7+, 4- | 13+, 4- |
| #3 | 1 | 21 | 20 (95%) | 11+ | 6+ | 2+, 9- | 1- | 2+1- |
| Inner | 2 | 43 | 33 (77%) | 19+ | 8+ | 14- | 1+, 1- | 7+, 1- |
| City | 3 | 70 | 58 (83%) | 28+, 2- | 12+, 1- | 6+, 20- | 1+, 5- | 12+, 1- |
| #4 | 1 | 24 | 24 (100%) | 3+, 10- | 22+, 1- | 1+ | 2+ | 7+, 3- |
| Rural | 2 | 19 | 8 (42%) | 3+ | 3+ |  | 1+ | 2+ |
|  | 3 | 25 | 22 (88%) | 7+ | 12+, 3- | 2- |  | 5+ |
| #5 | 1 | 25 | 19 (76%) | 11+, 0- | 7+, 3- | 10- | 3+ | 2+ |
| Urban | 2 | 51 | 47 (92%) | 27+, 2- | 23+, 4- | 9- | 4- | 5+ |
|  | 3 | 45 | 38 (84%) | 20+ | 10+ |  |  |  |
| #6 | 1 | 50 | 42 (84%) | 26+, 3- | 13+, 4- | 2+, 15- | 3+, 5- | 9+, 1- |
| Inner | 2 | 75 | 71 (95%) | 40+, 5- | 31+, 3- | 1+, 24- | 3+, 4- | 10+ |
| City | 3 | 80 | 66 (82%) | 28+, 4- | 22+, 5- | 1+, 16- | 4+, 5- | 9+, 3- |
|  |  |  | Ave. 85% |  |  |  |  |  |

(+) refers to positive statements, what did you like most? (-) refers to negative statements, what did you like least?
